# Year-Long Phenotypical Study of Calves Derived From Different Assisted-Reproduction Technologies

**DOI:** 10.3389/fvets.2021.739041

**Published:** 2022-01-10

**Authors:** Jordana S. Lopes, Cristina Soriano-Úbeda, Evelyne París-Oller, Sergio Navarro-Serna, Analuce Canha-Gouveia, Lucía Sarrias-Gil, José Joaquin Cerón, Pilar Coy

**Affiliations:** ^1^Physiology of Reproduction Group, Department of Physiology, Faculty of Veterinary, University of Murcia, Murcia, Spain; ^2^Institute for Biomedical Research of Murcia, IMIB-Arrixaca, Murcia, Spain; ^3^Interdisciplinary Laboratory of Clinical Analysis (Interlab-UMU), Faculty of Veterinary, University of Murcia, Murcia, Spain

**Keywords:** assisted reproductive technologies (ART), *in vitro* embryo production (IVP), cattle, biochemical and hematological studies, reproductive fluids

## Abstract

Assisted reproductive technologies play a major role in the cattle industry. An increase in the use of *in vitro*-derived embryos is currently being seen around the globe. But the efficiency and quality of the *in vitro*-derived embryos are substandard when compared to the *in vivo* production. Different protocols have been designed to overcome this issue, one of those being the use of reproductive fluids as supplementation to embryo culture media. In this study, *in vitro*-derived calves produced with reproductive fluids added to their embryo production protocol were followed for the first year of life pairwise with their *in vivo* control, produced by artificial insemination (AI), and their *in vitro* control, produced with standard supplementation in embryo production. The objective was to assess if any differences could be found in terms of growth and development as well as hematological and biochemical analytes between the different systems. All the analysed variables (physical, hematological, and biochemical) were within physiological range and very similar between calves throughout the entire experiment. However, differences were more evident between calves derived from standard *in vitro* production and AI. We concluded that the use of reproductive fluids as a supplementation to the embryo culture media results in calves with closer growth and development patterns to those born by AI than the use of bovine serum albumin as supplementation.

## Introduction

The current data on bovine embryo transfer (ET) illustrate how substantial the use of *in vitro*-produced (IVP) embryos has emerged in detriment of *in vivo*-derived (IVD) embryos, with a difference of almost double the number of IVP embryos being transferred (1). However, the ability of this method to produce a high number of embryos per oocyte is still suboptimal (2, 3). As so, recent alternatives to embryo culture medium composition or supplementation (4–7) have been proposed in order to improve the quality and quantity of blastocysts. These alternatives are capable of producing *in vitro* embryos in higher numbers (4, 6) with improved gene expression patterns (5, 7, 8) or with increased survival to cryopreservation (4, 5, 7) when compared to the standard IVP media. However, the medium- to long-term benefits of such changes remain largely unknown, as most studies were solely performed during the *in vitro* development period without follow-up to assess effects on pregnancy, calving, and offspring. When the IVP-derived calves were studied, differences in the phenotype of these animals were reported. Initially, one of the main IVP-related issues was the high incidence of abnormal offspring syndrome (9, 10). This was later found to be related to the *in vitro* coculture system or supplementation with serum (11). Yet, other differences in the phenotype of IVP-derived animals such as increased birth weight (12–16), increased growth performance at juvenile age (13, 17), or decreased milk yield (13) have been reported.

Most of the studies in the IVP-derived large animals have been focused on collecting data from IVP-derived embryos regarding conception rates, parturition easiness, consequences for the dam, birth weight, neonatal mortality, adult reproductive function, or even lactation performance (13, 14, 18). Other experiments have investigated criteria such as body weight and height during the first days of life (19, 20) or, less commonly, during the first year of life (21, 22). Very few studies have investigated long-term effects relating to general health parameters such as body temperature, heart rate, or respiratory rate (14, 17, 20, 23). There are also limited data exploring more specific health parameters such as general biochemical (17, 20, 23) or hematological analytes (17). Unfortunately, the published data are derived from measurements made during a short period of the calves' lives.

At this point, a main goal to achieve is to understand if the animals generated by IVP are indeed similar to those produced *in vivo*, not only at birth, but during growth and development until adulthood. As it was noted before in cattle (13) and other species (24), the conditions in which the embryos are set to during the preimplantation timeline are crucial to determine their future characteristics. If the changes in culture media could in fact influence their development later in life, then it could be of interest to analyse their full potential.

In the present study, we aimed to compare the changes in physiological variables and hematological and biochemical analytes in the development of calves from ET of improved IVP embryos [culture media supplemented with reproductive fluids (RF)] vs. calves from ET of control IVP embryos [standard culture media, bovine serum albumin (BSA)] and calves from artificial insemination (AI, *in vivo* control) while maintaining the same bull father throughout the groups. We hypothesized that calves born after being produced *in vitro* in our improved culture media would present similar phenotypical traits than the ones born after AI. Pregnancy outcomes have been reported previously (25), but here we focused on growth rates, physiological variables, and the values of hematological and biochemical analyses in several time points until 1 year of age of these calves.

## Materials and Methods

All chemicals were purchased from Sigma-Aldrich Chemical Company (Madrid, Spain) unless otherwise specified.

### Ethical Approval

The study was carried out following the Spanish Policy for Animal Protection RD 53/2013, which meets European Union Directive 2010/63/UE on animal protection. The Ethics Committee of Animal Experimentation of the University of Murcia and the Animal Production Service of the Agriculture Department of the Region of Murcia (Spain) (ref. no. A132141002) approved the procedures performed in this work.

### Embryo Production, Embryo Transfer, and Artificial Insemination

Data regarding embryo production, ET, and AI were already disclosed in Lopes et al. (25). Briefly, IVP embryos were produced after *in vitro* maturation and *in vitro* fertilization (IVF) in standard conditions of oocytes from slaughterhouse-obtained ovaries (crossbred Charolais and/or Limousine). After IVF, embryos were distributed into two groups: synthetic oviductal fluid (SOF) medium containing BSA as supplementation (BSA group) and SOF medium containing reproductive fluids (oviductal and uterine fluids, RF group) as supplementation. To follow the physiological event (26, 27), during the first 4 days of culture, embryos from the RF group were put in SOF medium supplemented with oviductal fluid (from early luteal phase of the estrous cycle, NaturARTs BOF-EL, Embryocloud, Spain) and the remaining days in SOF medium supplemented with uterine fluid (from mid-luteal phase of the estrous cycle, NaturARTs BUF-ML, Embryocloud, Spain) (5). Embryos on their days 7 and 8 were vitrified and stored until transfer. Recipient dams (dairy, Holstein) from a commercial farm (El Barranquillo SL, Torre Pacheco, Murcia, Spain) were synchronized, and ET was performed on days 6, 7, and 8 after estrus, receiving one embryo each. AI was performed on synchronized cows (dairy, Holstein) using the same bull as the one used for IVP embryos (Asturian Valley breed) (25). All the cows had the same feeding and housing conditions; parities varied between 1 and 7 (mean parity was 2.16).

### Animals

Animals were born at a commercial farm (El Barranquillo SL, Torre Pacheco, Murcia, Spain), separated immediately after birth from their dams and placed in separated pens. Colostrum (prepared from a pool of colostrum of different cows) was fed twice daily during the first 3 days of life, followed by milk replacer (Sprayfo Royal 60, Trouw Nutrition Sloten, Netherlands) also twice daily. Calves were born in the period between August 2018 and May 2019, transported to the Veterinary Farm at the University of Murcia (Spain) ~6 days post-birth and continued the same feeding plan. Water was available *ad libitum* as well as hay. At 15 days of life, calf starter was available *ad libitum* as well as forage. At 45 days of life, milk replacer was reduced to one feed daily, and at 60 days, it was terminated (weaning age = 60 days), continuing the calf starter until month 3. From 3 to 6 months of age, calves ate a grain mix plus hay, and from 6 months onward, grain mix and straw. Calves that were stillbirths or did not survive the first 60 days of age were excluded from the present data. Seven calves from AI group (5 males and 2 females), seven calves from BSA group (4 males and 3 females), and five calves from RF group (4 males and 1 female) were included in this year-long study. All the animal handling was performed by the same group of people (at both farms).

### Physical Parameters

Starting from birth until 1 year of age, calves were evaluated for the following health and growth criteria: body weight (kg) measured with a weight scale (BR 15, Baxtran, Giropes SL, Girona, Spain), body length (cm, distance from the head to the base of the tail measured with a tape measure), height at withers (cm, measured with a tape measure), thorax circumference (cm, measured with a tape measure), body temperature (°C, measured with a rectal thermometer), heart rate (beats per min, assessed with stethoscope), and respiratory rate (RPM, breaths per min, assessed with a stethoscope). Time points in days were: 0 (before colostrum intake), 3, 7, and 15 and every 15 days until day 360. Data from days 225, 315, and 345 were excluded due to unavailability to perform the examination in five or more calves. Average daily weight gain (ADWG) was calculated by subtracting the body weight of the previous assessment and dividing by the days passed between dates. The 1-year ADWG was calculated by subtracting the birth weight to the body weight at 1 year of age and dividing by 360 days. Body mass index was calculated by weight (kg)/withers^2^ (m) at each day.

### Blood Collection and Analysis

Blood samples were collected from jugular vein (calves <6 months of age) or caudal vein (calves >6 months of age) using lithium heparin vacutainer tubes (BD Vacutainer, BD Spain). Glucose (mg/dL) was evaluated immediately using test strips (GlucoMen LX, GlucoMen UK), and hematological analysis was performed in whole blood using a hematology analyser (Siemens ADVIA^®^ 120, USA). Parameters included in this study were as follows: hematocrit (%), erythrocytes (×10^6^ cells/μL), hemoglobin (g/dL), mean corpuscular volume (MCV; fL), mean corpuscular hemoglobin (MCH; g/dL), mean cell hemoglobin concentration (MCHC; g/dL), cell hemoglobin concentration mean (CHCM; g/dL), red blood cell distribution width (RDW; %); leukocytes (×10^3^ cells/μL), neutrophils (×10^3^ cells/μL), lymphocytes (×10^3^ cells/μL), monocytes (×10^3^ cells/μL), eosinophils (×10^3^ cells/μL), basophils (×10^3^ cells/μL), platelets (×10^3^ cells/μL), mean platelet volume (MPV; fL), plateletcrit (PCT; %), platelet distribution width (PDW; %), mean platelet component (MPC; g/dL), mean platelet mass (MPM; pg), large platelets (Large PLT; ×10^3^ cells/μL), reticulocyte hemoglobin content (CHr; pg), reticulocytes (×10^6^ cells/μL), and mean corpuscular volume of reticulocytes (MCVr; fL). After hematological analysis, blood was centrifuged at 1,008 g for 10 min, and plasma was stored at −80°C for biochemical analysis. Biochemical analysis was performed using an automatic chemistry analyser (Olympus AU400, Japan). Parameters included were as follows: total protein (g/dL), albumin (g/dL), globulin (g/dL), creatinine (mg/dL), urea (mg/dL), cholesterol (mg/dL), triglycerides (mg/dL), amylase (UI/L), lipase (UI/L), creatinine kinase (CK, UI/L), alkaline phosphatase (ALP; UI/L), gamma-glutamyl transpeptidase (GGT; UI/L), aspartate aminotransferase (AST; UI/L), alanine aminotransferase (ALT; UI/L), and total bilirubin (mg/dL). Data collection for glucose and hematological samples occurred during the following days: 0 (before colostrum intake), 3, 7, and 15 and every 15 days until day 90, followed by every 30 days until day 180 and every 60 days until day 360. Biochemical samples were analysed in the same days as hematological samples, excluding days 75, 90, and 150 of life.

### Statistical Analysis

Physical and blood parameters might be different between males and females, so a bivariate correlation (Pearson) was used with a significance level of *p* < 0.05. Sex was not statistically correlated to any of the parameters, so, calves were treated regardless of the sex. Data normality was assessed by Shapiro–Wilk test. Data were analysed using a mixed-effects model (when there were missing values) or repeated-measures ANOVA, followed by Tukey's multiple comparison test with an adjusted *p*-value. Day and group were considered fixed effects, and *p*-value was considered significant when < 0.05. Correlations between weight × creatinine and weight × protein were assessed using a Spearman correlation with a significance level of *p* < 0.05. Data are presented as mean ± standard error of the mean (SEM). Sample size for this experiment was calculated using a probability of error α = 0.05, a power of 95% (1-β), statistical test ANOVA (repeated measures), with three different groups and 12 measurements (the lowest number of our analysis, corresponding to the biochemical analysis). The required total estimated sample size was 15, but for convenience, we used all the available animals (*n* = 19). The statistical software used was GraphPad Prism version 8.0.0 (GraphPad Software, San Diego, CA, USA).

### Experimental Design

**Figure 1:**
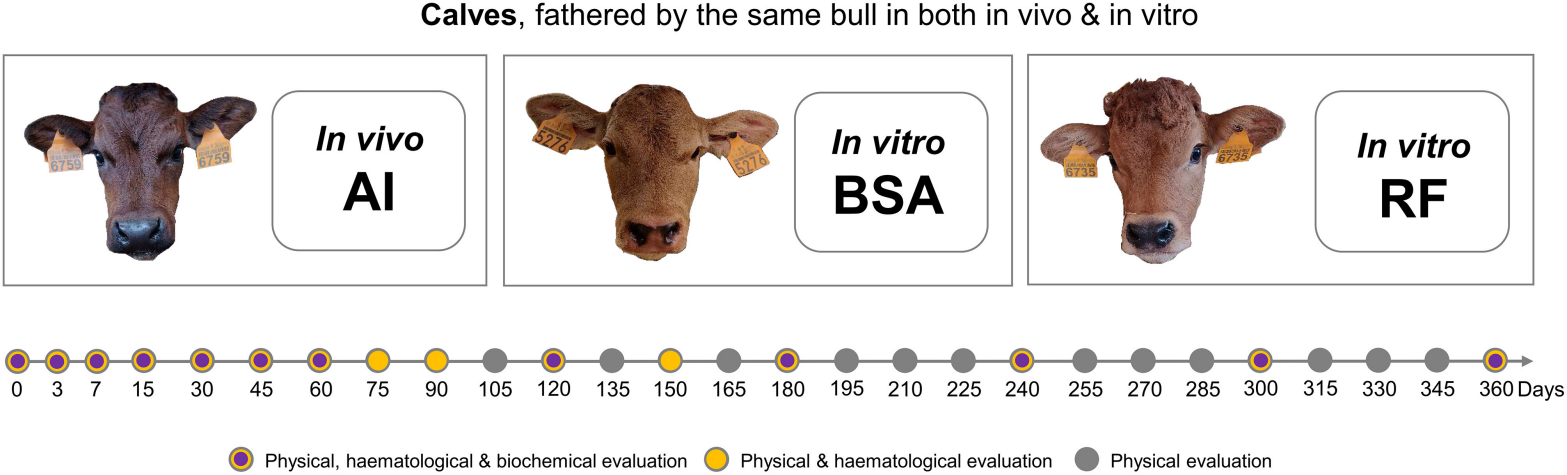
Representation of the experimental design with the different groups and time points (in days post-birth) of evaluation of each set of parameters. Calves, fathered by the same bull, were either *in vivo* derived from artificial insemination (AI), *in vitro* derived from embryos cultured with bovine serum albumin (BSA), or *in vitro* derived from embryos cultured with reproductive fluids (RF). Each calf was examined in specific days for physical, hematological, and/or biochemical parameters, depending on the day post-birth.

Calves born by AI (AI group) and by *in vitro* production with BSA or reproductive fluids as supplementation to embryo culture media (respectively, BSA and RF groups) were fathered by the same bull, followed closely throughout the first year of life. Physical examinations and blood collections for hematological and biochemical analyses took place in different time points, as detailed in Figure 1.

## Results

### Physical Variables

**Figure 2:**
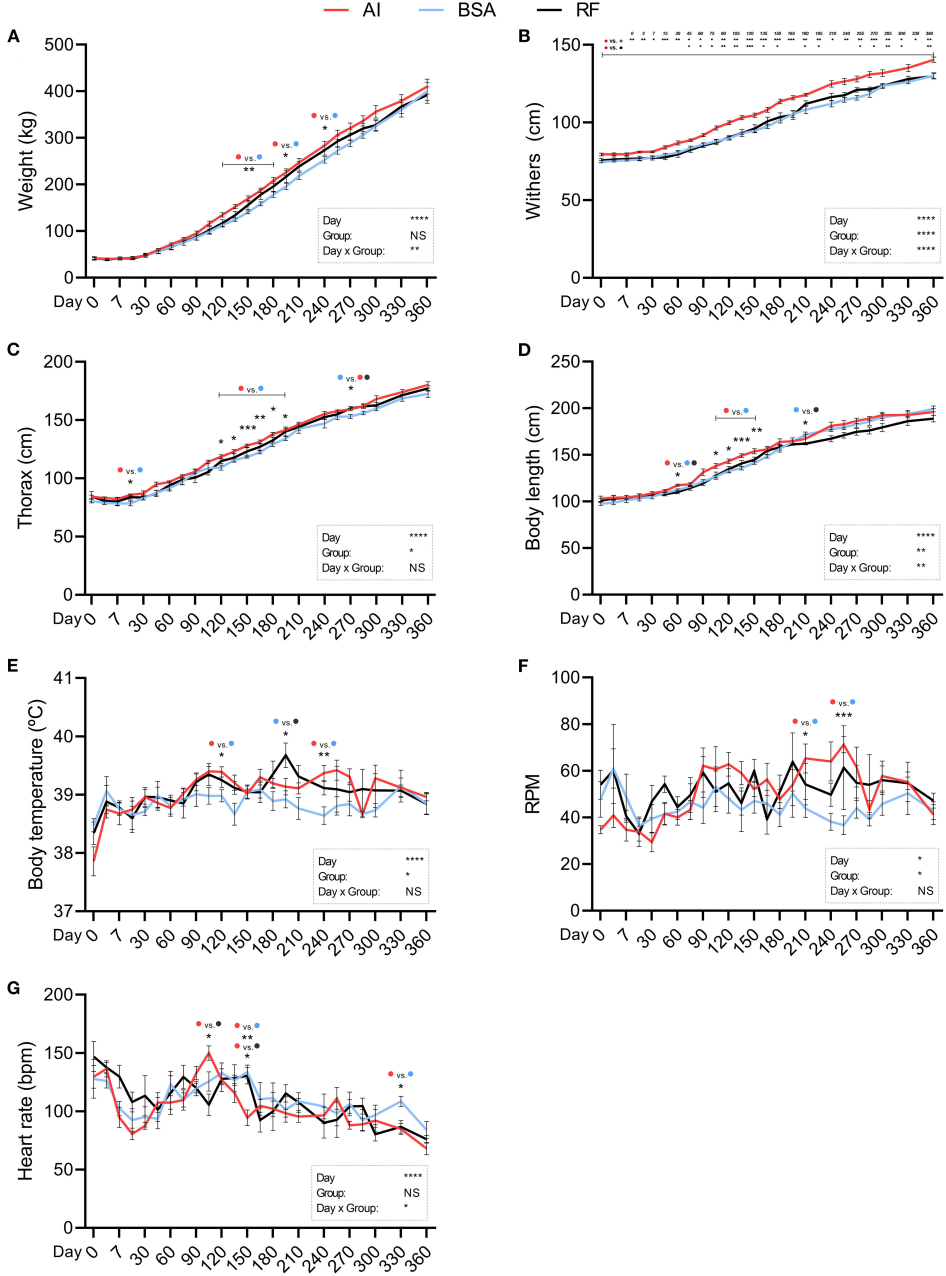
Physical parameters obtained during the first year of life from calves born after artificial insemination (AI), *in vitro* produced with bovine serum albumin (BSA), and *in vitro* produced with reproductive fluids (RF). Values represent mean ± SEM. Variables included: body weight [**(A)**, weight] in kg, height at withers [**(B)**, withers] in cm, thorax circumference [**(C)**, thorax) in cm, body length **(D)** in cm, body temperature **(E)** in °C, respiratory rate [**(F)**, RPM] in respirations per minute, and heart rate **(G)** in beats per minute. Asterisk(s) represent statistical difference and above the asterisk(s), the difference found is represented by color (AI, red; BSA, blue; RF, black). *p < 0.05; **p < 0.01; ***p < 0.001; ****p < 0.0001; NS, not significant.

**Figure 3:**
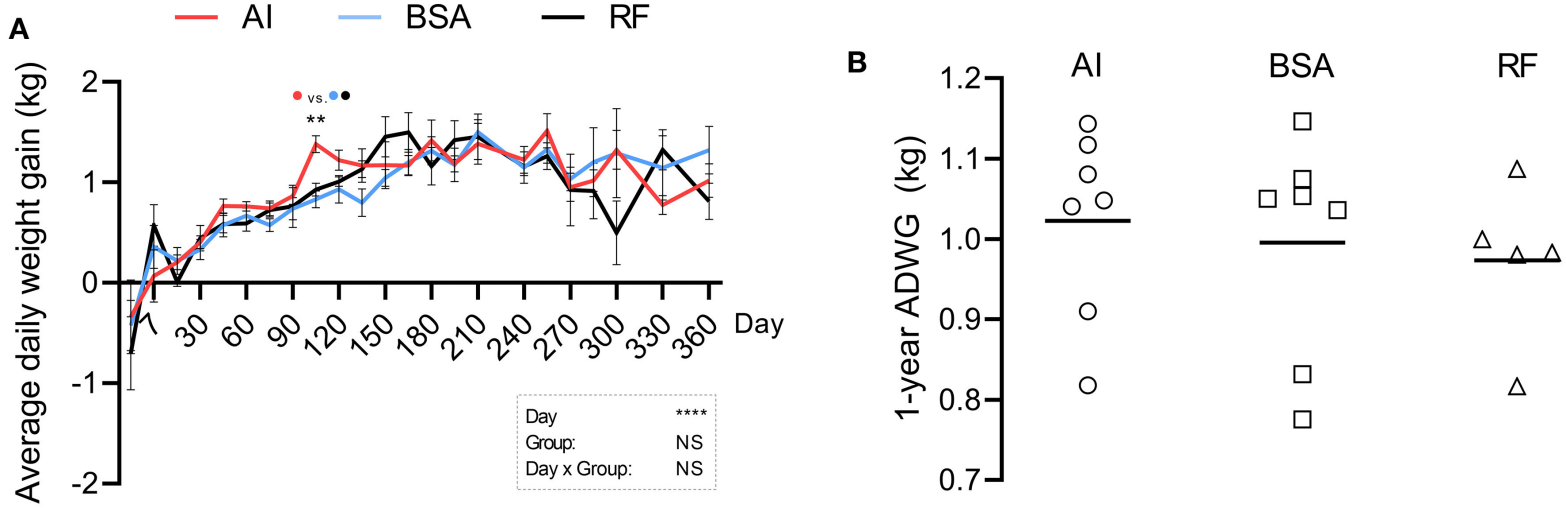
Calculated average daily weight gain (ADWG) of the first year of life from calves born after artificial insemination (AI), *in vitro* produced with bovine serum albumin (BSA), and *in vitro* produced with reproductive fluids (RF). In panel **(A)** values represent mean ± SEM. In panel **(B)** each symbol represents the total average weight gain of an individual, with the line representing the mean value for the group. Asterisks represent statistical difference, and above the asterisks, the difference found is represented by color (AI, red; BSA, blue; RF, black). **p < 0.01; ****p < 0.0001; NS, not significant.

**Figure 4:**
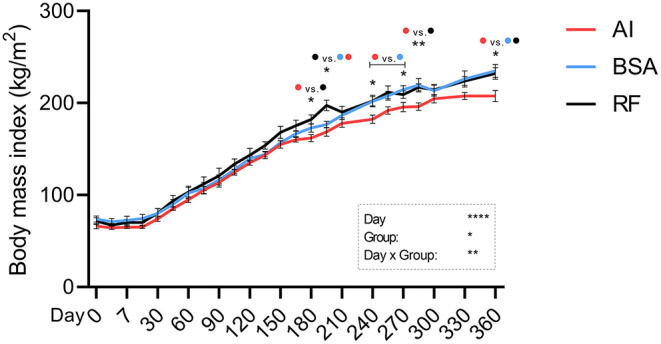
Calculated body mass index (kg/m^2^) during the first year of life from calves born after artificial insemination (AI), *in vitro* produced with bovine serum albumin (BSA), and *in vitro* produced with reproductive fluids (RF). Values represent mean ± SEM. Asterisk(s) represent statistical difference, and above the asterisks, the difference found is represented by color (AI, red; BSA, blue; RF, black). *p < 0.05; **p < 0.01; ****p < 0.0001; NS, not significant.

Body weight (Figure 2A) was significantly altered by the day of collection and day × group interaction. AI calves were heavier than BSA calves from days 120 to 195 and again on day 240, where AI calves weighed 284.00 ± 8.81 and BSA calves weighed 252.93 ± 6.89. Withers (Figure 2B) was affected by the day, group, and day × group interaction. AI calves were significantly taller than BSA calves from birth (79.43 ± 0.90 for AI and 74.71 ± 0.68 for BSA) to 1 year of age (140.43 ± 1.74 for AI and 130.21 ± 1.88 for BSA). AI calves were only taller than RF calves from days 45 to 150, then 180–195, and again on days 255–300, and lastly on day 360, where RF calves showed a mean value of 129.80 ± 1.69. Thorax circumference (Figure 2C) was conditioned by the day of collection and the group. AI calves presented a greater girth on day 15 (85.79 ± 0.94) in comparison to BSA calves (78.73 ± 1.95). From days 120 to 195, AI calves showed a larger chest circumference than BSA calves. On day 270, BSA calves had 153.14 ± 1.67, significantly shorter than AI calves (159.29 ± 0.84) and RF calves (160.00 ± 1.82). Body length (Figure 2D) was influenced by the day, group, and day × group interaction. When comparing AI calves with BSA calves, AI calves presented a significantly longer body than BSA calves on day 60 (117.43±1.13 vs. 112.43±1.07, respectively) and from days 105 to 150. RF calves showed shorter length of the body on day 60 (110.20 ± 1.91) when compared to AI calves, and then on day 210 (162.00 ± 1.23), shorter than BSA calves (171.86 ± 2.67). Body temperature (Figure 2E) was affected by the day of collection and influenced by the group: temperature was higher for AI calves vs. BSA calves on days 120 and 240 (39.39 ± 0.09 and 39.37 ± 0.13 vs. 38.98 ± 0.14 and 38.64 ± 0.15, respectively) and higher too for RF calves (39.68 ± 0.21) vs. BSA calves on day 195 (38.92 ± 0.15). Respiratory rate (Figure 2F) was affected by the day of collection along with the group, having at days 210 and 255 AI calves higher breaths per min (65.29 ± 6.22 and 71.20 ± 3.44) when compared to BSA calves (43.86 ± 3.91 and 36.67 ± 4.01). Heart rate (Figure 2G) was highly influenced by the day of measurement and day × group interaction: on day 105, AI calves had 150.00 ± 6.15 beats per min, significantly higher than RF calves with 105.60 ± 9.17. On day 150, AI calves showed significantly lower heart rate (94.43 ± 6.74) in comparison with those in both RF (130.50 ± 7.09) and BSA calves (133.71 ± 5.89). On day 330, AI calves showed inferior heart rate (84.80 ± 4.63) than BSA calves (108.50 ± 4.37). ADWG was altered by the day of data collection, but not by group, being the only difference found on day 105, where AI calves showed a higher weight gain than those in both BSA and RF calves (Figure 3A). The year-old ADWG was not significantly different between groups (Figure 3B). The body mass index was affected by the day of data collection, the group, and the day × group interaction: AI calves showed a lower index when compared to BSA calves on days 240, 270, and 360 and lower index when compared to RF calves on days 180, 195, 285, and 360. Additionally, the body mass index was also lower for BSA calves compared to RF calves on day 195 (Figure 4).

### Hematological Parameters

**Figure 5:**
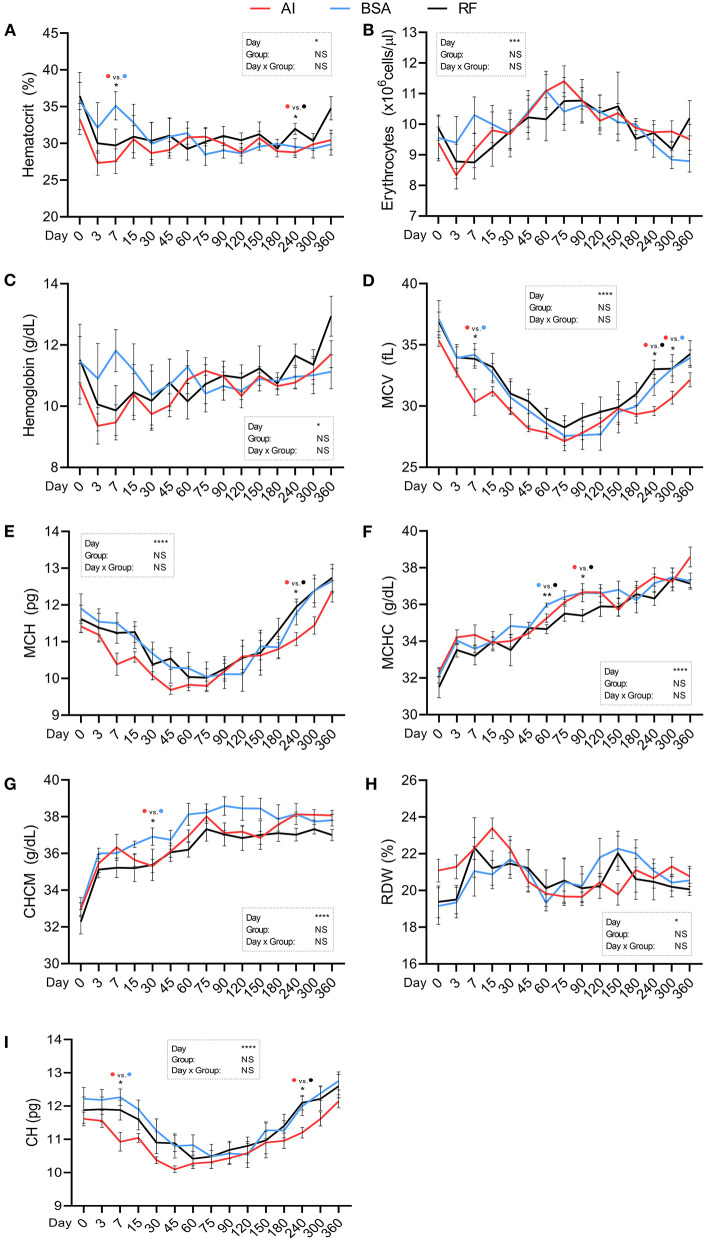
Hematological parameters (red blood cells) obtained during the first year of life from calves born after artificial insemination (AI), *in vitro* produced with bovine serum albumin (BSA), and *in vitro* produced with reproductive fluids (RF). Values represent mean ± SEM. Parameters included: hematocrit [**(A)**, %], erythrocytes [**(B)**, ×10^6^ cells/μL], hemoglobin [**(C)**, g/dL], mean corpuscular volume [**(D)**, MCV, fL], mean corpuscular hemoglobin [**(E)**, MCH, pg], mean cell hemoglobin concentration [**(F)**, MCHC, g/dL], cell hemoglobin concentration mean [**(G)**, CHCM, g/dL], red blood cell distribution width [**(H)**, RDW, %], and cellular hemoglobin [**(I)**, CH, pg]. Asterisk(s) represent statistical difference and above the asterisk(s), the difference found is represented by color (AI, red; BSA, blue; RF, black). *p < 0.05; **p < 0.01; ***p < 0.001; ****p < 0.0001; NS, not significant.

Red blood cells parameter values (Figure 5) were all influenced by the day of collection. Hematocrit (Figure 5A; %) was similar between groups, with two exceptions: one on day 7 where AI calves showed lower values (27.57 ± 1.76) vs. BSA calves (35.12 ± 1.92) and another on day 240 where AI calves showed lower values (28.79 ± 0.71) vs. RF calves (31.98 ± 0.76). Erythrocytes, hemoglobin, and RDW (Figures 5B,C,H, respectively) showed no significant differences between groups. MCV (Figure 5D; fL) was lower in AI calves on days 7 and 300 (30.31 ± 1.08 and 30.69 ± 0.53) compared to BSA calves (34.20 ± 0.92 and 33.06 ± 0.60, respectively) and on day 240 where AI calves had 29.60 ± 0.37 vs. RF calves that had 33.06 ± 1.14. MCH (Figure 5E; pg) was lower for AI calves vs. RF calves but only on day 240 (11.07 ± 0.18 vs. 11.95 ± 0.21. respectively). MCHC (Figure 5F; g/dL) was significantly lower for RF calves vs. BSA calves on day 60 (34.66 ± 0.25 vs. 35.97 ± 0.11, respectively) and vs. AI calves on day 90 (35.40 ± 0.33 vs. 36.67 ± 0.12, respectively, RF vs. AI). CHCM (Figure 5G; g/dL) was higher for BSA calves (36.91 ± 0.47) vs. AI calves (35.31 ± 0.36) on day 30. CH (Figure 5I; pg) was lower for AI calves when compared to BSA calves on day 7 (10.93 ± 0.28 vs. 12.27 ± 0.25) and when compared to RF calves on day 240 (11.20 ± 0.16 vs. 12.22 ± 0.36, respectively, AI vs. RF).

**Figure 6:**
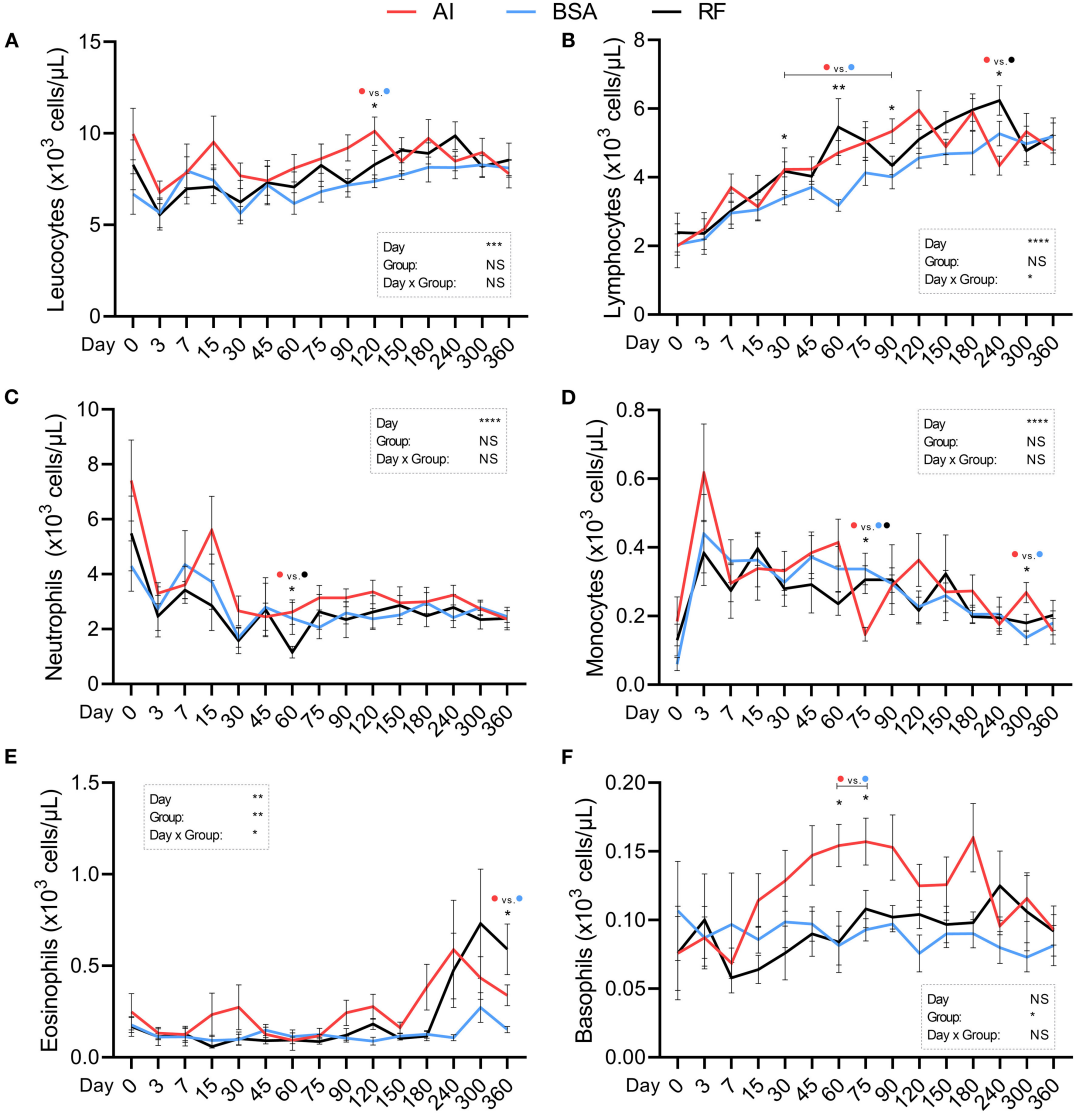
Hematological parameters (white blood cells) obtained during the first year of life from calves born after artificial insemination (AI), *in vitro* produced with bovine serum albumin (BSA), and *in vitro* produced with reproductive fluids (RF). Values represent mean ± SEM. Parameters included: leukocytes [**(A)**, ×10^3^ cells/μL], lymphocytes [**(B)**, ×10^3^ cells/μL], neutrophils [**(C)**, ×10^3^ cells/μL], monocytes [**(D)**, ×10^3^ cells/μL], eosinophils [**(E)**, ×10^3^ cells/μL], and basophils [**(F)**, ×10^3^ cells/μL]. Asterisk(s) represent statistical difference, and above the asterisk(s), the difference found is represented by color (AI, red; BSA, blue; RF, black). *p < 0.05; **p < 0.01; ***p < 0.001; ****p < 0.0001; NS, not significant.

White blood cell parameters were all influenced by the day of collection with the exception of basophil concentration. Leukocyte concentration (Figure 6A; ×10^3^ cells/μL) was higher for AI calves (10.12 ± 0.77) in comparison to BSA calves (7.38 ± 0.35) on day 120. Lymphocyte concentration (Figure 6B; ×10^3^ cells/μL) was influenced by the day × group interaction, and significant differences were found on days 30, 60, and 90, where AI calves had higher values (4.23 ± 0.22, 4.72 ± 0.35, and 5.34 ± 0.36) than those in BSA calves (3.41 ± 0.21, 3.18 ± 0.17, and 4.01 ± 0.35) and again on day 240, where AI calves had lower values (4.34 ± 0.28) than those in RF calves (6.24 ± 0.43). Neutrophil concentration (Figure 6C; ×10^3^ cells/μL) was higher for AI calves (2.62 ± 0.45) in relation to RF calves (1.16 ± 0.21) on day 60. Monocyte concentration (Figure 6D; ×10^3^ cells/μL) was significantly different on days 75 and 300: AI calves showed lower values on day 75 vs. both groups (0.15 ± 0.02 vs. 0.34 ± 0.05 and 0.31 ± 0.04, respectively, AI vs. BSA and RF); on day 300, concentration was higher for AI calves (0.27 ± 0.03) vs. BSA calves (0.14 ± 0.02). Eosinophil concentration (Figure 6E; ×10^3^ cells/μL) was affected by the group and the day × group interaction being only significantly different on day 360, where BSA calves showed lower values (0.151 ± 0.02) than AI calves (0.34±0.06), and although that difference was also present vs. RF calves (0.59 ± 0.14), it was only a tendency (p = 0.07). Basophils concentration (Figure 6F; ×10^3^ cells/μL) was influenced by the group, and two significant differences were found: AI calves had higher values on days 60 and 75 (0.15 ± 0.02 and 0.16 ± 0.02, respectively) than those in BSA calves (0.08 ± 0.01 and 0.09 ± 0.01).

**Figure 7:**
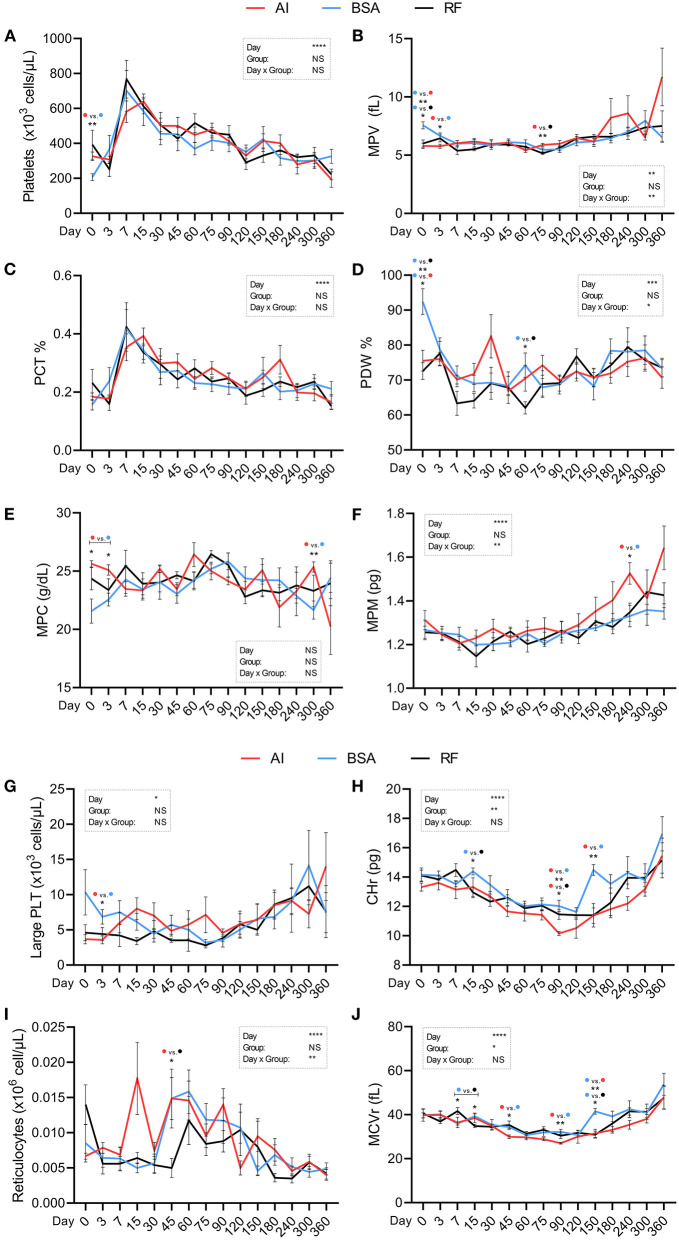
Hematological parameters (platelet and reticulocyte parameters) obtained during the first year of life from calves born after artificial insemination (AI), *in vitro* produced with bovine serum albumin (BSA), and *in vitro* produced with reproductive fluids (RF). Values represent mean ± SEM. Parameters included: platelets [**(A)**, ×10^3^ cells/μL], mean platelet volume [**(B)**, MPV, fL], platelet ratio [**(C)**, PCT, %], platelet distribution width [**(D)**, PDW, %], mean platelet component [**(E)**, MPC, g/dL], mean platelet mass [**(F)**, MPM, pg], large platelets [**(G)**, Large PLT, ×10^3^ cells/μL], reticulocyte hemoglobin content [**(H)**, CHr, pg], reticulocytes [**(I)**, ×10^6^ cells/μL], mean corpuscular volume of reticulocytes [**(J)**, MCVr, fL]. Asterisk(s) represent statistical difference, and above the asterisk(s), the difference found is represented by color (AI, red; BSA, blue; RF, black). *p < 0.05; **p < 0.01; ***p < 0.001; ****p < 0.0001; NS, not significant.

Platelet and reticulocyte parameters (Figure 7) were significantly influenced by the day of collection, except for MPC. Platelet count (Figure 7A; ×10^3^ cells/μL) at birth was 206.50 ± 19.76 for BSA calves, while AI calves showed a mean value of 326.50 ± 24.33. MPV (Figure 7B; fL), besides being influenced by the day × group interaction, showed several significant differences: BSA calves showed higher values than those in the other two groups at birth (7.57 ± 0.31 vs. 5.8 ± 0.23 for AI and 6.0 ± 0.31 for RF) and also higher than those in AI calves on day 3 (6.66 ± 0.25 vs. 5.77 ± 0.17, respectively, BSA and AI); on day 75, RF calves showed lower values (5.16 ± 0.12) than those in AI calves (5.89 ± 0.13). PCT (Figure 7C) did not show any significant difference among groups. PDW (Figure 7D; %) was affected by the day × group interaction and showed higher values for BSA (92.45 ± 3.70) calves than those in the other two groups on day 0 (75.53 ± 2.99 and 72.58 ± 2.40, respectively, AI and RF) and higher again on day 60, with a mean value of 74.28 ± 3.5, but only vs. RF group (62.05 ± 1.76). MPC (Figure 7E; g/dL) had higher values for AI calves when compared to those in BSA calves on days 0 (25.63 ± 0.28 vs. 21.57 ± 1.04), 3 (25.10 ± 0.45 vs. 22.56 ± 0.55), and 300 (25.36 ± 0.44 vs. 21.66 ± 0.77). MPM (Figure 7F; pg) showed one significant difference on day 240, where AI calves had 1.53 ± 0.05 and BSA calves had 1.33 ± 0.05, and the parameter was altered by the day × group. Large PLT (Figure 7G; ×10^3^ cells/μL) were higher for BSA calves than AI calves on day 3 (6.86 ± 1.03 vs. 3.57 ± 0.57, BSA and AI, respectively) with no further differences. CHr (Figure 7H; pg) values were also influenced by the group, having higher values in BSA calves (14.39 ± 0.24) than those in RF calves (12.96 ± 0.37) on day 15 and then higher values in both BSA and RF calves than those in AI calves on day 90 (12.03 ± 0.38, 11.46 ± 0.34 vs. 10.16 ± 0.14, respectively), adding again higher values for BSA vs. AI calves on day 150 (14.49 ± 0.37 vs. 11.37 ± 0.52). Reticulocyte count (Figure 7I; ×10^6^ cells/μL) was affected by the day × group interaction but only showed a significant difference on day 45, with AI calves having higher values (0.015 ± 0.003) than RF calves (0.005 ± 0.001). MCVr (Figure 7J; fL) was influenced by the group, with several significant differences: on day 7, BSA calves had lower values vs. RF calves (35.95 ± 0.64 vs. 41.66 ± 1.56), but on day 15, it was the opposite (39.30 ± 1.04 vs. 34.86 ± 0.78, respectively); on days 45, 90, and 150, BSA calves showed higher values (34.39 ± 0.99, 32.24 ± 1.11, and 41.51 ± 1.28) in comparison with those in AI calves (29.97 ± 0.79, 27.04 ± 0.38, 31.50 ± 1.87) but only higher than those in RF calves on day 150 (30.90 ± 1.60).

### Biochemical Parameters

**Figure 8:**
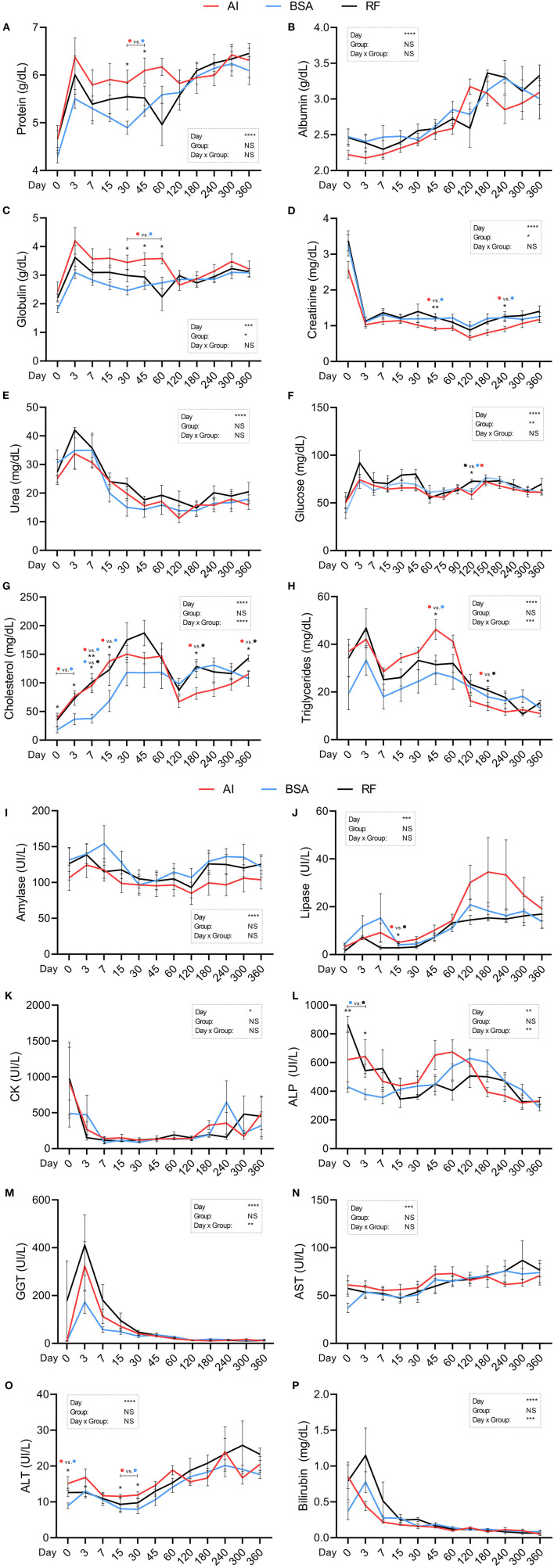
Biochemical parameters obtained during the first year of life from calves born after artificial insemination (AI), *in vitro* produced with bovine serum albumin (BSA), and *in vitro* produced with reproductive fluids (RF). Values represent mean ± SEM. Parameters included: total protein [**(A)**, g/dL], albumin [**(B)**, g/dL], globulin [**(C)**, g/dL], creatinine [**(D)**, mg/dL], urea [**(E)**, mg/dL], glucose [**(F)**, mg/dL], cholesterol [**(G)**, mg/dL], triglycerides [**(H)**, mg/dL], amylase [**(I)**, UI/L], lipase [**(J)**, UI/L], creatinine kinase [**(K)**, CK, UI/L], alkaline phosphatase [**(L)**, ALP, UI/L], gamma-glutamyl transpeptidase [**(M)**, GGT, UI/L], aspartate aminotransferase [**(N)**, AST, UI/L], alanine aminotransferase [**(O)**, ALT, UI/L], and total bilirubin [**(P)**, mg/dL]. Asterisk(s) represent statistical difference, and above the asterisk(s), the difference found is represented by color (AI, red; BSA, blue; RF, black). *p < 0.05; **p < 0.01; ***p < 0.001; ****p < 0.0001; NS, not significant.

All biochemical parameters were significantly affected by the day of collection (Figures 8A–P). Total protein (Figure 8A; g/dL) showed a higher concentration in AI calves on days 30 and 45 (5.84 ± 0.26 and 6.10 ± 0.26, respectively) when compared to BSA calves (4.90 ± 0.14 and 5.25 ± 0.12, respectively). Albumin (Figure 8B) and urea (Figure 8E) did not differ across groups. Globulin (Figure 8C; g/dL), on the other hand, was also altered by the group, having higher values in AI calves on days 30, 45, and 60 (3.45 ± 0.26, 3.56 ± 0.23, and 3.58 ± 0.18, respectively) when compared to those in BSA calves (2.47 ± 0.15, 2.63 ± 0.13, and 2.74 ± 0.20, respectively). Creatinine (Figure 8D; mg/dL) had a group influence, and significant differences were found between AI calves on days 45 (0.91 ± 0.05) and 60 (0.91 ± 0.08) and BSA calves (1.20 ± 0.06 and 1.22 ± 0.08, respectively). Glucose (Figure 8F; mg/dL) was affected by the group, but only one difference was significant on day 120, where RF calves had 75.5 ± 4.32, while AI calves showed 65.2 ± 4.70 and BSA calves had 69.39 ± 2.30. Total cholesterol (Figure 8G; mg/dL) behaved differently on a day × group interaction: BSA calves had 17.91 ± 5.35 on day 0, 37.74 ± 7.57 on day 7, and 68.45 ± 18.62 on day 15, which were significantly lower than those in AI calves on days 0 and 15 (38.50 ± 3.40 and 138.11 ± 10.11, respectively) and lower than those in RF calves on day 7 (101.34 ± 14.86). Additionally, AI calves showed lower values when compared to RF calves on days 180 and 360 (81.66 ± 10.34 and 115.52 ± 5.74 vs. 128.59 ± 8.22 and 143.37 ± 5.36, respectively). Triglycerides (Figure 8H; mg/dL) were affected by a group × day interaction, being the values for AI calves higher on day 45 vs. BSA calves (46.23 ± 4.16 vs. 28.12 ± 4.95, respectively), but lower than those in RF calves on day 180 (13.91 ± 1.65 vs. 20.63 ± 1.68, respectively). Amylase, CK, and AST (Figures 8I,K,N) did not show any alterations between groups. Lipase concentration (Figure 8J; UI/L) was higher for AI calves (5.07 ± 0.54) vs. RF (2.82 ± 0.27) on day 15, with no further significant differences. ALP concentration (Figure 8L; UI/L) was influenced by the day × group interaction, and particularly on days 0 and 3, BSA calves showed a lower concentration (430.43 ± 35.82 and 377.86 ± 35.49) than RF calves (865.85 ± 56.70 and 543.40 ± 44.79). GGT and bilirubin (Figures 8M,P) were both affected by day × group, but no significant differences were found among groups. AI calves showed higher ALT concentration (Figure 8O; UI/L) on days 0 (15.16 ± 1.92), 15 (11.54 ± 0.55), and 30 (11.91 ± 0.86) when compared to BSA calves (8.96 ± 0.78, 8.07 ± 1.06, and 7.91±1.19, respectively).

### Correlations Between Physical and Biochemical Data

**Figure 9:**
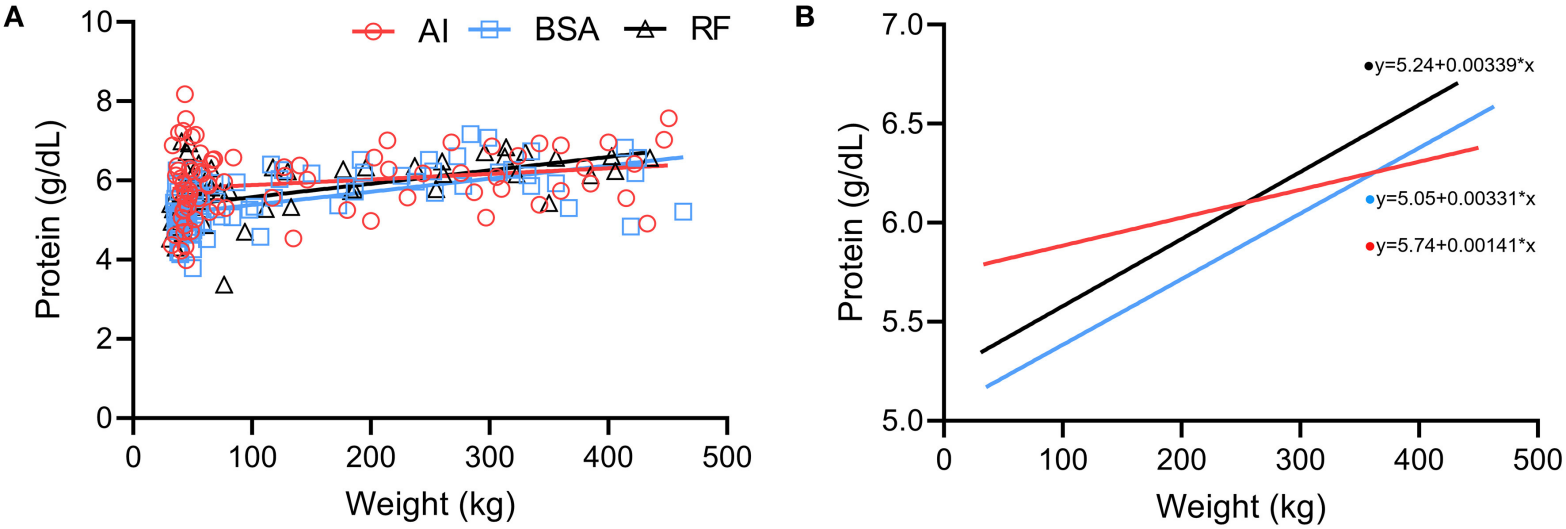
Comparison between weight (kg) and protein concentration (g/dL) obtained during the first year of life from calves born after artificial insemination (AI), *in vitro* produced with bovine serum albumin (BSA), and *in vitro* produced with reproductive fluids (RF). **(A)** Each symbol represents one individual value, where red circles represent AI calves; blue squares, BSA calves; and black triangles, RF calves—the lines represent the linear regression. **(B)** Simple linear regression of the values obtained in panel **(A)**, where red represents AI calves; blue, BSA calves; and black, RF calves.

A positive correlation between weight and protein (Figure 9A) was present in all groups (AI *r* = 0.23 with *p* = 0.0375; BSA *r* = 0.59 with *p* < 0.0001; RF *r* = 0.60 with *p* < 0.0001). The simple linear regression lines (Figure 9B) show that the slopes were significantly (p < 0.05) different: even though all groups increased their total protein as their weight increased, the AI group had a different pattern where the increase was not so pronounced as the BSA and RF groups (AI *r*^2^ = 0.05; BSA *r*^2^ = 0.32; RF *r*^2^ = 0.29).

**Figure 10:**
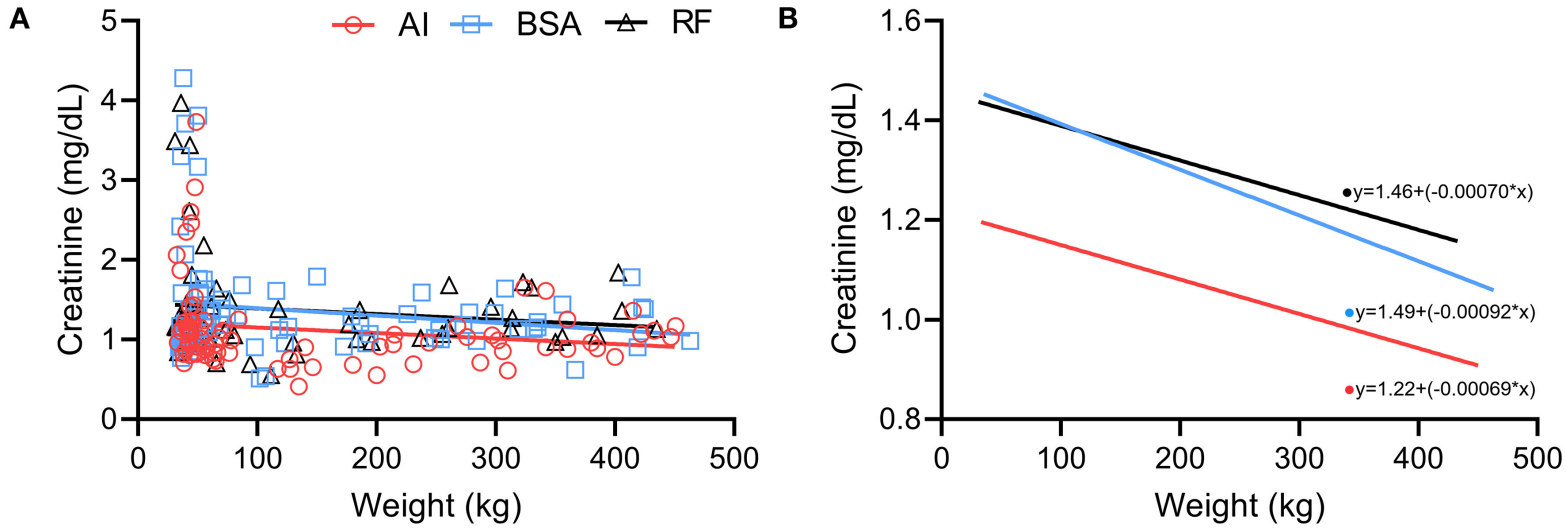
Comparison between weight (kg) and creatinine concentration (mg/dL) obtained during the first year of life from calves born after artificial insemination (AI), *in vitro* produced with bovine serum albumin (BSA), and *in vitro* produced with reproductive fluids (RF). **(A)** Each symbol represents one individual value, where red circles represent AI calves; blue squares, BSA calves; and black triangles, RF calves—the lines represent the linear regression. **(B)** Simple linear regression of the values obtained in panel **(A)**, where red represents AI calves; blue, BSA calves; and black, RF calves.

A negative correlation between weight vs. creatinine (Figure 10A) was present in AI calves, but no correlation was found on BSA and RF calves (AI *r* = −0.24 with *p* = 0.0304; BSA *r* = 0.03 with *p* > 0.10; RF *r* = −0.13 with *p* > 0.10). When performing a simple linear regression (Figure 10B), the regression lines behave similarly, but AI calves show significantly lower levels of creatinine per kg (p < 0.05) when compared to BSA and RF calves (AI *r*^2^ = 0.03; BSA *r*^2^ = 0.03; RF *r*^2^ = 0.02).

## Discussion

The continuous increase of the use of IVP-ET systems in the cattle industry brings the necessity of a better understanding of whether calves born through this combination are indeed similar to those born after AI or natural mating. Many disadvantages (28) and advantages (11) of the use of IVP embryos have been noted before, but it is not yet totally clear if the differences found in early stages are maintained throughout the growth and development of these animals, nor when do they disappear. In dairy cattle, authors have reported differences in milk production (13), but little is known about female or male beef calves, since most studies are limited in time. Here, we describe the changes occurring during the maturity of these animals, males and females, conceived by different reproductive technologies until they reach 1 year of age with special incidence on biochemical and hematological analysis, since they could flag alterations that could be invisible to phenotypical evaluation.

Body weight of the calves was not different, with some exceptions happening between days 120 and 240 where AI calves were significantly heavier than BSA calves. The weaning weight, which in our case occurred on day 60, was also not different between groups, being in accordance with some authors (13) and in disagreement with others (17) who reported higher weight at the same age for IVP-derived calves. Interestingly, both referenced studies show values at around 80 kg for IVP calves, which were much heavier calves than ours, either explained by differences in genetics or feeding plans. The ADWG is a parameter that, in theory, would help us understand if calves from a specific group would have a faster growth/weight gain. Although the day was determinant in those gains, we noticed only one significant difference on day 105. That means that all our calves, regardless of the group, developed similarly. When analysing the year-long ADWG differences were not found. Our values were similar to those reported to happen in female and male Asturian Valley (29). This could be reasoned by the fact that we mixed females and males–due to low number of females available—but also a difference in breeds. We did not find any difference in the development of our males vs. females, as they showed similar weight/height values.

As for birth weight, it was not different between groups, being in disagreement with some studies (10, 13, 17) and in agreement with others (20, 21) when comparing AI with IVP-derived calves. The birth weight also did not define the weight in adulthood. McEvoy et al. (12) suggested that the birth weight should be used as a factor when evaluating the postnatal development of calves derived by IVP. When analysing our data on IVP calves (BSA and RF groups) and separating the calves by their birth weight (low, normal, or heavy), the ADWG did not show any difference between them (data not shown). On the contrary, when calculating the differences concerning the actual weight/day of the calves, we observe that the group does make a difference, particularly up to 120 days, but no differences at that point and onward. The ADWG is critical to understand if the feeding was appropriate to each animal, which in our study was indeed. However, McEvoy et al. (12) found differences in the heart size of the bulls, suggesting that there is more to the evaluation of growth and development than simply the body weight. More data are needed within our study regarding these calves, as their organs might also differ in weight as it happened in the study of McEvoy et al. (12).

Jacobsen et al. (30) found no differences in terms of body length and withers between IVP calves derived by different culture systems and their AI controls. In our study, the bull-father was the same for all calves. However, since AI calves had Holstein-mother and IVP calves had cross-Beef-mother, we found several differences in height at withers, thorax circumference, and body length. These differences could easily be explained by their different mother-genetics, as Holstein Frisian is a taller breed than Asturian Valley, thus justifying the deepest thorax and higher height. Body length had fewer differences and was especially located around the major growth period (between 60 and 210 days), when the ADWG was also higher for AI calves.

Data of the remaining health-related physical parameters investigated in this study for IVP calves are controversial. Sangild et al. (31) found increased respiratory rate at birth for IVP calves when compared to that in AI, but although we did see the same pattern (higher rate in IVP-derived calves at birth, BSA and RF groups), our difference was not significant. Rérat et al. (17) found no differences in respiratory rate at birth [as others (14)] nor at 112 days, though these authors found higher respiratory rate for AI calves than that in IVP at the latter age. Heart rate was found to be lower in IVP-derived calves by some authors at birth (14), but we did not find that difference, as in accordance to others (23). Body temperature was also found to be similar at birth between calves derived from different reproductive techniques (23), as it also happened in our study. In general, we found some occasional differences in these health-related physical parameters between calves. Considering that they all still fit the standard-normal values, it is most likely that these differences may have been influenced by the stress of each calf that may influence respiratory and heart rate and/or the seasonal temperature, since some calves were born in different seasons, meaning that the time they reached each age was measured with different outdoor temperatures. The evaluation of all these physical and health parameters allowed us to determine if the speed of development was accompanied by an apparent healthy body system.

Regarding hematological and biochemical data, it can be said that, in general, the red blood cells, reticulocytes, white blood cells, and platelets did not show changes of clinical significance between groups, and their values are in agreement with those reported in healthy individuals by others for calves and adult cattle (31–35). Among the analytes that showed significant differences between groups of higher magnitude, we found that total protein, globulin, creatinine, cholesterol, triglycerides, and ALT were different during the preweaning period. These analytes showed higher values in AI calves compared to those in BSA calves with the exception of creatinine, which was lower for AI calves when compared to BSA calves. All the animals received the same amounts and pools of colostrum and the same milk replacement, independently of their origin, and therefore, these results cannot be compared to a difference in intake or quality of colostrum/feeding. We could only hypothesize that there might be a difference in the metabolism of these animals, especially noted in the pre-weaning period, but without further investigation, we cannot confirm it. However, all the studied analytes were similar between groups at the end of the experimental period, indicating that the different systems of production of embryos might not affect the biochemical profiles of the animals in adult life. The analysis of all these blood parameters comes as an aid to assess if there was a sign of disruption of any system of the body of the calves that might pass unseen to the physical examination.

As for the interpretation of the correlations found between protein and weight and between creatinine and weight, the different patterns observed in the AI animals might reflect their genetic differences, and even though AI calves started to develop with higher protein and creatinine concentrations, they still could not reach the same muscular production as those derived from beef × beef genetics (both BSA and RF groups). Because of these genetic impacts, future projects should only focus on one genetic line to reduce the variation that genes may have on growth and development.

In general, we found that the IVP-derived calves reached the same stage of growth and development in adulthood as their AI counterparts. This is a conclusion that does not agree with many previous authors. Recently, a long-term study with rabbits on the effects of vitrification and ET (both techniques that we used with our IVP calves) showed that, at birth, those animals were heavier than the ones produced by AI, but the growth performance until adulthood was poorer, resulting in adult rabbits with less weight (36). Likewise, in mice, Fernandez-González et al. (24) showed that solely using different culture media may result in different phenotypes like increased weight during growth or alterations in behavior or memory tests. Siqueira et al. (13) with a large study reported not only increased birth weight for IVP-derived calves in comparison with AI but also increased weight at first breeding. Duranthon and Chavatte-Palmer (37) provided an extensive review on the long-term effects of different reproductive technologies, showing how the techniques we use may influence future outcomes. Those differences begin in early stages, as it has been noted in preimplantation embryos, where different techniques may lead to imprinted methylation errors (38, 39) or distinct patterns of global methylation (40, 41). With more than 7 million children born all over the world derived from assisted reproduction, and many millions more in the animal field, the differences found are not astonishing enough to prevent the use of these techniques. We must—as well as we should—always have in mind that each single one current technique may be improved. Thus, the reason why we decided to use reproductive fluids in our embryo culture media. Despite bovine serum albumin being a major source of protein capable of allowing embryo development *in vitro*, a lot of other proteins, messengers, and cytokines are missing, and this may be one of the reasons why the IVP yield is still low. Post-transfer, the results on pregnancy rates of IVP embryos are also lower than those of IVD embryos (42). By adding the reproductive fluids (oviductal and uterine) at the same pace of embryo development, the goal was to better mimic the natural environment. Therefore, trying to compensate those lower outcomes. Even though it did not increase embryo yield (25.9% with RF vs. 26.7% with BSA) nor pregnancy rates (both 22.2% at day 30) (25), the calves born from this system were more similar to those born by AI than the BSA-derived calves. In a global analysis, we had more significant differences between AI-derived calves and BSA-derived calves than we had between AI and RF or BSA and RF. This could mean that RF-derived calves were indeed reprogrammed to be closer to what *in vivo* growth may be, but further analysis—particularly of their methylation patterns *in vivo*—needs to be taken to better support this hypothesis. Limitations of this study are clearly related to the fact that we used different breeds in conceiving our calves and, of course, the number of animals obtained that influenced the sex ratio, not allowing the separation of data by sex. The increase in this number would be of interest, since it would allow the study of reproductive parameters of the adult animals.

In conclusion, the use of reproductive fluids as a supplementation to the embryo culture media results in calves with closer growth and development patterns to those born by AI than the use of BSA as supplementation.

## Data Availability Statement

The data that support the findings of this study are available from the corresponding author, PC, upon reasonable request.

## Ethics Statement

The animal study was reviewed and approved by the Ethics Committee of Animal Experimentation of the University of Murcia and the Animal Production Service of the Agriculture Department of the Region of Murcia (Spain) (Ref. No. A132141002) following the Spanish Policy for Animal Protection RD 53/2013, which meets European Union Directive 2010/63/UE on animal protection.

## Author Contributions

PC designed the project. PC and JL decided on the methodology. JL, PC, and JC analyzed the data and wrote the first draft of the article. All authors contributed to data collection, reviewed, and approved the final version of the article.

## Funding

This work was supported by the European Union, Horizon 2020 Marie Skłodowska-Curie Action (Rep-Biotech 675526); Spanish Ministry of Economy and Competitiveness (MINECO) & European Regional Development Fund (FEDER), (AGL2015-66341-R); Fundación Séneca, Agencia de Ciencia y Tecnología de la Región de Murcia (20040/GERM/16); and I+D+i PID2020-113366RB-I00, funded by MCIN/AEI/10.13039/501100011033/ and FEDER “Una maneira de hacer Europa”.

## Conflict of Interest

The authors declare that the research was conducted in the absence of any commercial or financial relationships that could be construed as a potential conflict of interest.

## Publisher's Note

All claims expressed in this article are solely those of the authors and do not necessarily represent those of their affiliated organizations, or those of the publisher, the editors and the reviewers. Any product that may be evaluated in this article, or claim that may be made by its manufacturer, is not guaranteed or endorsed by the publisher.
